# Effect of General vs. Regional Anesthesia on Mortality, Complications, and Prognosis in Older Adults Undergoing Hip Fracture Surgery: A Propensity-Score-Matched Cohort Analysis

**DOI:** 10.3390/jcm12010080

**Published:** 2022-12-22

**Authors:** Guolei Zhang, Huihui Chen, Junpu Zha, Jingtao Zhang, Jun Di, Xiaoqing Wang, Xin Hu, Xin Xu, Junfei Guo

**Affiliations:** 1 Department of Orthopaedics Surgery, Third Hospital of Hebei Medical University, Shijiazhuang 050051, China; 2 Orthopaedic Institute of Hebei Province, Shijiazhuang 050051, China; 3 Department of Nephrology, Fourth Hospital of Hebei Medical University, Shijiazhuang 050011, China; 4 Department of Biochemistry and Molecular Biology, College of Basic Medicine, Hebei Medical University, Shijiazhuang 050011, China; 5 Key Laboratory of Neural and Vascular Biology of Ministry of Education, Shijiazhuang 050011, China; 6 Key Laboratory of Medical Biotechnology of Hebei Province, Hebei Medical University, Shijiazhuang 050011, China

**Keywords:** anesthesia, hip fracture, older adults, mortality, complications, functional outcomes, propensity score matching, cohort

## Abstract

The choice of the type of anesthesia (TOA) used in hip fracture surgery in older adults is still controversial. The main question is not whether regional anesthesia (RA) or general anesthesia (GA) is superior, but in which patients the type of anesthesia may affect the outcome after surgery. In this retrospective analysis of surgically treated intertrochanteric fracture patients, we used propensity score matching (PSM) to investigate whether clinically relevant differences in outcomes were observed in mortality, complications, and functional outcomes between RA and GA. After screening 2934 consecutive patients, 2170 were ultimately included, including 841 in the GA group and 1329 in the RA group. After PSM, 808 remained in each group. Patients receiving GA were more prone to have a shorter duration for their operation and higher total hospital costs than patients with RA (*p* = 0.034 and 0.004, respectively). We also observed that the GA group has a higher rate of pulmonary complications, while the RA group has a higher rate of cardiac complications (*p* = 0.017 and 0.011, respectively). No significant difference was observed in mortality, functional outcomes, and other complications (all *p* > 0.05). The clinical innovation of this study was the potential value of GA for patients with cardiac diseases and of RA for patients with pulmonary diseases.

## 1. Introduction

Fractures can be detrimental for older adults as they are at an increased risk for death, disability, and loss of independence [1,2,3,4]. Meanwhile, with the aging of society, the absolute number of fractures increases, especially hip fractures, which are one of the most common types of lower limb fractures in older adults [5]. Annually, there are over 1 million hip fractures worldwide, a number that is particularly high in developing nations. A report has been made that there will be more than 4 million hip fractures worldwide, and 1.5 million of them will occur in China by the year 2050 [6]. Nowadays, intertrochanteric fractures (IF), which are low-energy fractures that are mostly related to osteoporosis, account for almost half of all hip fractures and represent a major health concern for older adults with multiple concurrent comorbidities who tend to have difficulty achieving and maintaining good health, placing a heavy medical and financial burden on society [7].

Even though frail patients have been managed in significant ways over the past few decades, there is still room for improvement. The type of anesthesia (TOA), as one modifiable risk factor affecting patient outcomes, is of great importance. However, the choice of TOA in hip fracture surgery in geriatric patients is still inconclusive [8,9,10,11,12]. The reason may be that previous studies tend to include all hip fracture patients regardless of their age, fracture type, and internal fixation type, which can complicate the analysis of the risks associated with anesthesia and mortality [4,12,13]. In addition, the majority of studies examined mortality after hip fracture surgery, whereas little attention has been directed to showing the effect of TOA on prognosis and complications involving multiple systems. Finally, the great variety in the length of the follow-up may have contributed to the inconsistent findings in different study populations; ultimately, it cannot be determined which of the TOAs yield a more favorable outcome or in which patients the TOA may condition the outcome after surgery [4,9,12].

Therefore, we used propensity score matching (PSM) to investigate whether clinically relevant differences in outcomes were observed in the present study, regarding mortality, complications, and functional outcomes between regional and general anesthesia in older adults who underwent IF surgery by proximal femoral nail anti-rotation (PFNA).

## 2. Materials and Methods

### 2.1. Study Design

Data were retrospectively collected by electronic medical records reviews, consisting of ED admission charts, electronic hospital records, and/or discharge data for all surgical patients to repair an IF in an urban, Level I regional trauma center that ranked among the national top 10. Patients who were admitted between January 2014 and May 2018, 65 years or older, injured from fall or high-energy injuries, with an admission delay of injury <48 h, who underwent hip surgery of closed reduction and internal fixation by PFNA and who received a minimum 2-year follow-up were included. Patients with multiple fractures or injuries, with pathological or open hip fractures, received conservative treatment, and patients with missing data were excluded. The research was conducted according to ethical principles. In compliance with the Declaration of Helsinki, the study was overseen and approved by the institution’s internal review board. In this observational study without intervention, consent was waived. To protect patient confidentiality, all collected patient data were recorded anonymously. This study focused primarily on the dichotomy between general anesthesia (GA) and regional anesthesia (RA), and the patients were divided into the GA group and RA group accordingly. The RA group was administered epidural anesthesia, spinal anesthesia, or the two combined, while the GA group included the patients who had planned GA and those who started with any type of RA but were converted to GA in the final stages of the procedure.

Data included patients’ demographics, including gender, age, body mass index (BMI), place of residence (rural or urban), as well as smoking and drinking history; injury-related data consisting of injury mechanisms, fracture type, and surgical delay; surgery-related data including health status as rated by the American Society of Anesthesiologists (ASA)(classified as I to VI) as well as mECM as has been described previously [1,14]; in-hospital data including Hb level at admission, numerical rating scores (NRS) at admission [15,16], Geriatric Depression Scale (GDS), and functional independence measures (FIM). Outcome analyses consisted of the duration of operation, intraoperative blood loss, requirement and amount for blood transfusion, length of hospital stay (LOS), total hospital charges (THC), and complications involving multiple systems. Following up with participants, the functional status, survival status, and death date were obtained. A follow-up period was defined as the date the participant entered the cohort, and an endpoint event as the date of the most recent follow-up visit, whichever occurred first. In addition, mortality and functional outcomes were measured at 30 days, 90 days, 180 days, 12 months, and 24 months and classified as independent walking, use of walking aids, wheelchair, bedridden, and death.

### 2.2. Statistical Analysis

Continuous variables were evaluated for normality by applying the Shapiro–Wilk test. Numerical variables satisfying normality were compared using Student’s *t*-test to obtain group mean differences, and data are presented as mean ± standard deviation (SD). Median and interquartile range (IQR) were reported as data were nonnormally distributed using the Mann–Whitney U test. Categorical variables are shown as proportions, and the differences were analyzed using chi-square or Fisher’s exact test.

PSM is a statistical technique that simulates the characteristics of a randomized controlled trial within an observational study design regardless of confounding factors. Therefore, to reduce selection bias and potential confounding factors, fifteen different covariates were used in the PSM model by using a 1:1 ratio via the caliper matching of 0.04: gender, age group, BMI group, residence, smoking status, drinking status, injury mechanism, fracture type, surgical delay, ASA, mECM, Hb level at admission, NRS, GDS, and FIM. After PSM, continuous variables were analyzed using paired t-tests, while categorical variables were analyzed using paired chi-square tests or Fisher’s exact test. All data analyses were performed using IBM SPSS Statistics for Windows, version 26.0 (IBM, Armonk, NY, USA). The level of significance was set at *p* < 0.05.

## 3. Results

**Figure 1:**
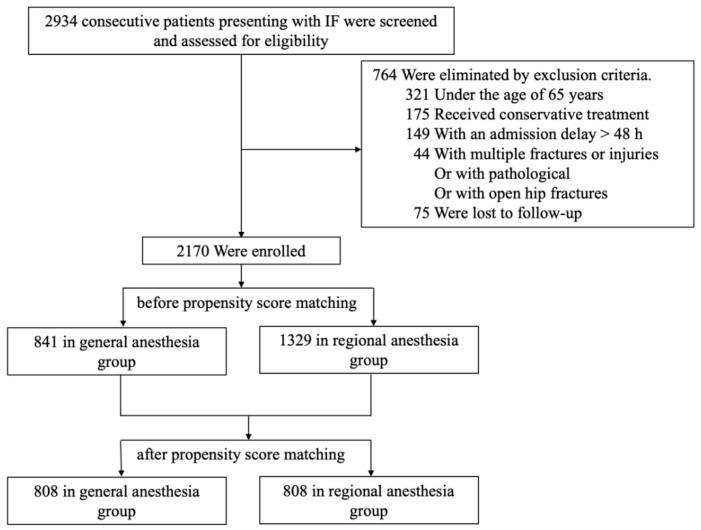
Flow diagram of included patients.

From January 2014 and May 2018, we identified 2934 consecutive patients admitted to our hospital. In total, 764 patients were excluded based on the exclusion criteria after being reviewed and assessed for eligibility. Finally, 2170 patients (including 841 in the GA group and 1329 in the RA group) met the inclusion and exclusion criteria and were included in the initial analysis, and a propensity score was calculated for each of them (Figure 1).

Of these, the majority of patients were women (69.3% in the GA group and 66.4% in the RA group, respectively), and a majority of admissions were in the 80–89 years of age (43.3%) range in the GA group, while most were in the 70–79 years (40.0%) of age range in the RA group. The prevalence of the various characteristics in the GA or RA cohorts is detailed in Table 1. Before PSM, baseline characteristics including BMI group, surgical delay, and Hb level at admission differed between the two groups. A total of 808 matched pairs existed after PSM, and this resulted in similar baseline characteristics among the two groups (all *p* > 0.05, Table 1).

The impact of RA compared with GA on the duration of operation, intraoperative blood loss, incidence and volume of blood transfusion, LOS, THC, mortality, complications, and functional outcomes before and after matching were shown in Table 2. Before PSM, statistical analysis revealed that RA was found to be protective for two complications including severe complications and hematological complications compared with GA. The difference, however, was not significant after PSM. Nevertheless, those patients receiving GA were more prone to have higher THC than patients with RA before and after PSM. Although the differences in the duration of operation were comparative between the two groups before matching, they differed after matching. Although a trend was observed among participants who received GA to have more intraoperative blood loss and blood transfusion volume and worse functional outcomes, mortalities rates at 30 days, 90 days, 180 days, 12 months, and 24 months, LOS, and complications including severe complications and hematological complications than those patients who received RA, the differences fell outside statistical significance after PSM (all *p* > 0.05, Table 2). Notably, our results identified that the GA group has higher rate of pulmonary complications (12.0% vs. 8.4%, *p* = 0.017), while the RA group has a higher rate of cardiac complications (23.0% vs. 17.9%, *p* = 0.011). At the end of the study, 167 (19.8%) patients in the GA group and 262 (19.8%) patients in the RA group had died. Table 2 showed there was no significant difference in all-cause mortality.

## 4. Discussion

According to the results of this study, patients receiving GA are prone to have higher THC and a shorter duration of operation than patients with RA. We also observed that the GA group has a higher rate of pulmonary complications, while the RA group has a higher rate of cardiac complications. No significant difference was observed in intraoperative blood loss, blood transfusion rate and volume, LOS, mortality, functional outcomes, and other complications (all *p* > 0.05). Our findings with regards to mortality parallel the results from a recent large-scale meta-analysis showing no difference in 30-day mortality with GA vs. RA [11]. Based on the other literature reviews and large-scale studies, our results are also in line with current evidence [10,12,17]. There are some indications from multicenter randomized trials that suggest better outcomes associated with RA than GA during the in-hospital, 30-day, or 90-day timeframe [8,9,18]. Several older studies by McLaren et al. (1978) [19], Valentin et al. (1986) [20], and Radcliff et al. (2008) [21] also supported RA for hip fracture surgeries. By contrast, Parker et al. found a higher mortality risk with RA after 1 year [22]. In the case of this completely different situation, it is not surprising given the confounding differences in study design, case volume, study locations, medical systems, and inclusion and exclusion criteria. Additionally, with the advancement in surgical techniques and anesthesia, the differences between different TOAs no longer existed. This can be manifested by the variations in mortalities, with a 30-day mortality <1.0% in our institution as compared with that of 6–8% in Radcliff’s and Valentin’s research. This is due, in part, to the particular management of elderly hip fracture patients who receive standard combination therapy in specialist geriatric trauma orthopedic wards in our hospital, which has been described in detail in our prior literature [23].

Despite notable progress having been made in understanding and managing perioperative complications, several controversial aspects remain regarding pneumonia, lower extremity deep venous thrombosis, and acute myocardial infarctions. Van Waesberghe et al. found that RA is a protective factor for acute myocardial infarctions, and the choice of TOA showed no statistical significance in the incidence of pneumonia [11]. In contrast, Luger et al. demonstrated a lower incidence of pneumonia and lower extremity deep venous thrombosis for RA and no difference in acute myocardial infarction [24]. According to our results, we observed that RA had a better effect than the GA inhibiting pulmonary complications, which agrees with several previous researchers [25,26]. This is because GA with intubation may further aggravate pulmonary disease and cause acute respiratory distress syndrome [27]. Conversely, our results also demonstrated that the RA group has a higher risk for cardiac complications. A possible explanation for this is that GA makes patients unconscious through using various intravenous or inhaled drugs, which causes fewer cerebrovascular accidents and a sshorter anesthesia time compared with local anesthesia to minimize the possible impact of anesthesia residues on patients [28,29]. To the best of our knowledge, in particular patients with high risks for hemodynamic deterioration (e.g., patients with severe aortic stenosis, heart failure, coronary heart disease) may benefit by GA that allow for controlling peripheral resistances better than RA. Nevertheless, several researchers reached the opposite conclusion, including prior large observational studies [9,30] and randomized controlled trials [22]. However, heterogeneity existed among these studies with regards to the fracture type, surgery, hospitals, and medical systems. Previous studies [26,31,32] have proven the marginal advantage of RA for the risk of hypercoagulable states and deep vein thrombosis compared with GA in patients with hip fractures. They emphasize that patients undergoing hip fracture surgery are mainly elderly people who usually suffer from cardiovascular diseases, diabetes, and other senile diseases, with a relatively slow blood flow and hypercoagulable blood due to long-term bed rest. Patients receiving GA with muscle relaxant causea decrease in prothrombin and activated partial thromboplastin times, increase in tissue factor, vWF, plasminogen activator inhibitor-1 (PAI-1), and tissue plasminogen activator, resulting in a hypercoagulable and hypofibrinolytic state postoperatively, ultimately arousing thrombosis [33,34]. Our data also indicated that patients receiving GA tended to have higher rates of hematological complications before PSM. However, the difference was small and did not reach statistical significance after PSM.

Further, among these complications possibly related to the TOA of hip fractures in elderly patients, delirium has been reported associated with an increased incidence of cognitive dysfunction, extended LOS, and mortality [14,35,36]. In the past, numerous studies have examined the role of TOA in delirium rates after hip fracture surgery, although the conclusions of these studies yet remain controversial. Ahn et al. [37] conducted a nationwide population-based study and demonstrated that RA was associated with better outcomes in terms of delirium, while other prospective or meta-analyses [38,39] showed no benefit of RA compared to GA. Concerning our results, we did not confirm this correlation. The most likely reason for this discrepancy is the lack of accuracy in defining delirium; thus, it caused clinical physicians to overlook it and made it difficult to evaluate [13]. This suggests that caution is required when interpreting the results of the effect of TOA on delirium so far, and additional investigations into specific types of anesthesia including more nuanced measures are recommended.

In contrast to previous studies, this study’s strength lies in the patients with a specialized fracture type following recent data demonstrating the same surgery using a single internal fixation, as well as a PSM approach to statistics, ruling out the effects of possible confounding factors. Other strengths are the inclusion of a greater number of variables for the assessment of confounding and sets of scoring systems than in most previous meta-analyses and administrative data studies. Another strength of our study is its longitudinal setting, where all participants were followed at multiple time points including 30, 90, 180 day, and 12- and 24-month mortality. In addition to the very short-term outcomes other researchers have reported, these findings represent important time extensions. As far as we know, this is the first study to comprehensively investigate the effect of TOA on many different aspects of adverse outcomes including not only mortality but also function outcomes and complications. The study is only retrospective and is a single-center observational study, which poses a weakness. Other factors, such as blood examination and patient education, should also be considered, which has been shown to have a consolidating and influencing relationship and relevance with clinical functions, as these may influence our findings [40,41].

## 5. Conclusions

The clinical innovation of this study was the potential value of GA for patients with cardiac diseases and of RA for patients with pulmonary diseases. Future high-quality and better-designed randomized controlled multi-center trials investigating all components of perioperative laboratory values, patient characteristics, and the surgical care pathway could be used to better clarify the issue of TOA in this patient population.

## Figures and Tables

**Table 1:** Comparisons of patient characteristics at baseline before and after propensity score matching.

Variables	Pre-Matching			Post-Matching		
	General Group (*n* = 841)	Regional Group (*n* = 1329)	*p* Value	General Group (*n* = 808)	Regional Group (*n* = 808)	*p* Value
Demographics						
Gender, *n* (%)			0.16			0.49
Male	258 (30.7%)	446 (33.6%)		248 (30.7%)	261 (32.3%)	
Female	583 (69.3%)	883 (66.4%)		560 (69.3%)	547 (67.7%)	
Age group, *n* (%)			0.06			0.44
65–69	94 (11.2%)	136 (10.2%)		85 (10.5%)	90 (11.1%)	
70–79	322 (38.3%)	532 (40.0%)		312 (38.6%)	342 (42.3%)	
80–89	364 (43.3%)	522 (39.3%)		352 (43.6%)	299 (37.1%)	
90–99	58 (6.9%)	134 (10.1%)		56 (6.9%)	77 (9.5%)	
≥100	3 (0.3%)	5 (0.4%)		3 (0.4%)	0 (0.0%)	
BMI, kg/m^2^, *n* (%)			0.001 *			0.09
Normal (BMI < 24)	504 (59.9%)	904 (68.0%)		497 (61.5%)	523 (64.7%)	
Overweight (24 ≤ BMI < 28)	266 (31.6%)	332 (25.0%)		249 (30.8%)	211 (26.1%)	
Obesity (BMI ≥ 28)	71 (8.5%)	93 (7.0%)		62 (7.7%)	74 (9.2%)	
Residence, *n* (%)			0.58			0.88
Rural	285 (33.9%)	466 (35.1%)		280 (34.7%)	283 (35.0%)	
Urban	556 (66.1%)	863 (64.9%)		528 (65.3%)	525 (65.0%)	
Smoking (Yes)	182 (21.6%)	294 (22.1%)	0.55	176 (21.8%)	196 (24.3%)	0.29
Drinking (Yes)	41 (4.9%)	45 (3.4%)	0.08	28 (3.5%)	38 (4.7%)	0.21
Injury-related data						
Injury mechanism, *n* (%)			0.26			0.63
Low energy	823 (97.9%)	1290 (97.1%)		790 (97.8%)	787 (97.4%)	
High energy	18 (2.1%)	39 (2.9%)		18 (2.2%)	21 (2.6%)	
Fracture type, *n* (%)			0.37			0.88
Stable (A1.1–A2.1)	464 (55.2%)	707 (53.2%)		442 (54.7%)	439 (54.3%)	
Unstable (A2.2–A3.3)	377 (44.8%)	622 (46.8%)		366 (45.3%)	369 (45.7%)	
Surgical delay, days	6 (4, 7)	5 (4, 7)	0.001 *	6 (4, 7)	6 (4, 7)	0.19
Surgery-related data						
ASA, *n* (%)			0.24			0.71
1	175 (20.8%)	239 (18.0%)		158 (19.6%)	164 (20.3%)	
2	244 (29.0%)	372 (28.0%)		236 (29.2%)	217 (26.9%)	
3	299 (35.6%)	489 (36.8%)		293 (36.2%)	292 (36.1%)	
4	107 (12.7%)	191 (14.4%)		105 (13.0%)	113 (14.0%)	
5	16 (1.9%)	38 (2.8%)		16 (2.0%)	22 (2.7%)	
mECM, *n* (%)			0.12			0.81
<0	12 (1.4%)	16 (1.2%)		12 (1.5%)	7 (0.9%)	
0	374 (44.5)	665 (50.0%)		364 (45.0%)	373 (46.2%)	
1–5	141 (16.8%)	210 (15.8%)		138 (17.1%)	132 (16.3%)	
6–13	261 (31.0%)	372 (28.0%)		246 (30.4%)	249 (30.8%)	
≥14	53 (6.3%)	66 (5.0%)		48 (6.0%)	47 (5.7%)	
In-hospital data						
Hb level at admission, g/dL			0.01 *			0.58
Hb ≥ 12	216 (25.7%)	417 (31.4%)		211 (26.1%)	219 (27.1%)	
12 > Hb ≥ 10	365 (43.4%)	531 (40.0%)		355 (43.9%)	335 (41.5%)	
10 > Hb ≥ 8	206 (24.5%)	327 (24.6%)		198 (24.5%)	216 (26.7%)	
Hb < 8	54 (6.4%)	54 (4.0%)		44 (5.5%)	38 (4.7%)	
NRS	5.3 ± 1.8	5.3 ± 1.7	0.70	5.3 ± 1.8	5.4 ± 1.7	0.25
GDS	4.1 ± 1.4	4.1 ± 1.4	0.97	4.1 ± 1.4	4.2 ± 1.5	0.35
FIM	83.3 ± 10.1	84.0 ± 10.4	0.18	83.6 ± 10.0	83.7 ± 10.5	0.81

Values are presented as the number (%) or the mean ± SD (standard deviation). * *p* < 0.05, statistical significance. BMI, body mass index; ASA, American Society of Anesthesiologists; mECM, modified Elixhauser’s Comorbidity Measure; NRS, numerical rating scores; GDS, Geriatric Depression Scale; FIM, functional independence measure.

**Table 2:** Patient outcomes analyses before and after propensity score matching.

Variables	Pre-Matching			Post-Matching		
	General Group (*n* = 841)	Regional Group (*n* = 1329)	*p* Value	General Group (*n* = 808)	Regional Group (*n* = 808)	*p* Value
Duration of operation, mins	97.9 ± 35.8	100.1 ± 34.9	0.16	97.7 ± 35.8	101.5 ± 35.9	0.03 *
Intraoperative blood loss, mL	200 [150, 300]	200 [100, 300]	<0.001 *	200 [100, 300]	200 [100, 300]	0.44
Blood transfusion (Yes)	652 (77.5%)	988 (74.3%)	0.09	623 (77.1%)	607 (75.1%)	0.35
Blood transfusion volume, mL	4 [2, 6]	2 [0, 4]	0.001 *	4 [3, 6]	4 [2.5, 5.5]	0.55
Length of hospital stay, days	13 [11, 17]	13 [10, 17]	0.07	13 [11, 17]	13 [10, 18]	0.53
Total hospital costs, yuan	43,126.6 ± 12,869.3	42,040.4 ± 12,057.1	0.05 *	43,125.3 ± 12,942.8	41,433.6 ± 10,741.0	0.004 *
Mortality (Yes)						
30-day mortality	4 (0.5%)	9 (0.7%)	0.55	4 (0.5%)	7 (0.9%)	0.36
90-day mortality	9 (1.1%)	15 (1.1%)	0.90	9 (1.1%)	10 (1.2%)	0.82
180-day mortality	20 (2.4%)	27 (2.0%)	0.59	20 (2.5%)	15 (1.9%)	0.39
12-month mortality	45 (5.4%)	59 (4.4%)	0.33	44 (5.4%)	35 (4.3%)	0.30
24-month mortality	90 (10.7%)	111 (8.4%)	0.07	89 (11.0%)	70 (8.7%)	0.11
Complications (Yes)						
Severe complications	167 (19.9%)	216 (16.3%)	0.03 *	160 (19.8%)	132 (16.3%)	0.07
Cardiac complications	177 (21.0%)	307 (23.1%)	0.26	145 (17.9%)	186 (23.0%)	0.01 *
Pulmonary complications	79 (9.4%)	144 (10.8%)	0.28	97 (12.0%)	68 (8.4%)	0.02 *
Neurological complications	73 (8.7%)	102 (7.7%)	0.40	63 (7.8%)	78 (9.7%)	0.19
Hematological complications	385 (45.8%)	550 (41.4%)	0.04 *	336 (41.6%)	370 (45.8%)	0.09
Endocrine/metabolic complications	600 (71.3%)	903 (67.9%)	0.10	576 (71.3%)	549 (67.9%)	0.14
Functional Outcomes (Yes)			0.61			0.16
Independent walking	338 (40.2%)	544 (40.9%)		321 (39.7%)	331 (41.0%)	
Use of walking aids	257 (30.6%)	420 (31.6%)		243 (30.1%)	265 (32.8%)	
Use of wheelchair	57 (6.8%)	68 (5.1%)		57 (7.1%)	35 (4.3%)	
Bedridden	22 (2.6%)	35 (2.6%)		22 (2.7%)	21 (2.6%)	
Death	167 (19.8%)	262 (19.8%)		165 (20.4%)	156 (19.3%)	

Values are presented as the number (%) or the mean ± SD (standard deviation) or median (interquartile range). * *p* < 0.05, statistical significance.

## Data Availability

All the data supporting the study findings are within the manuscript. Additional detailed information and raw data are available from the corresponding author (Junfei Guo) on reasonable request.

## Abbreviations

IF: intertrochanteric fracture; mECM: modified Elixhauser comorbidity method; TOA: type of anesthesia; PSM: propensity score matching; PFNA: proximal femoral nail antirotation; GA: general anesthesia; RA: regional anesthesia; BMI: body mass index; ASA: American Society of Anesthesiologists; NRS: numerical rating scores; GDS: Geriatric Depression Scale; FIM: functional independence measure; LOS: length of hospital stay; THC: total hospital costs; ADL: activities of daily living; SD: standard deviation; IQR: interquartile range.
